# EGFRvIII Promotes Cell Survival during Endoplasmic Reticulum Stress through a Reticulocalbin 1-Dependent Mechanism

**DOI:** 10.3390/cancers13061198

**Published:** 2021-03-10

**Authors:** Juliana Gomez, Zammam Areeb, Sarah F. Stuart, Hong P. T. Nguyen, Lucia Paradiso, Ahmad Zulkifli, Sonakshi Madan, Vijay Rajagopal, Magdalene K. Montgomery, Hui K. Gan, Andrew M. Scott, Jordan Jones, Andrew H. Kaye, Andrew P. Morokoff, Rodney B. Luwor

**Affiliations:** 1 Department of Surgery, The University of Melbourne, The Royal Melbourne Hospital, Parkville, VIC 3050, Australia; JulianaG@student.unimelb.edu.au (J.G.); z.areeb@student.unimelb.edu.au (Z.A.); sstuart@student.unimelb.edu.au (S.F.S.); hong.nguyen1@unimelb.edu.au (H.P.T.N.); lucia.paridso@unimelb.edu.au (L.P.); ahmadz@student.unimelb.edu.au (A.Z.); madans@student.unimelb.edu.au (S.M.); jordan.jones@mh.org.au (J.J.); a.kaye@unimelb.edu.au (A.H.K.); morokoff@unimelb.edu.au (A.P.M.); 2 Cell Structure and Mechanobiology Group, Department of Biomedical Engineering, Melbourne School of Engineering, The University of Melbourne, Parkville, VIC 3010, Australia; vijay.rajagopal@unimelb.edu.au; 3 Department of Physiology, The University of Melbourne, Parkville, VIC 3010, Australia; magdalene.montgomery@unimelb.edu.au; 4 Olivia Newton-John Cancer Research Institute, La Trobe University, Heidelberg, VIC 3084, Australia; hui.gan@onjcri.org.au (H.K.G.); andrew.scott@onjcri.org.au (A.M.S.); 5 Department of Neurosurgery, The Royal Melbourne Hospital, Parkville, VIC 3050, Australia; 6 Department of Neurosurgery, Hadassah Hebrew University Medical Centre, Jerusalem 91120, Israel

**Keywords:** EGFRvIII, RCN1, ER stress, apoptosis, glioblastoma

## Abstract

**Simple Summary:**

A key molecule, EGFRvIII has been shown to provide several growth advantages for brain tumors. However, we have found a new mechanism in which the EGFRvIII provides increased survival to brain cancer cells when under sub-optimal conditions. Specifically, we have found that the EGFRvIII drives the expression of a molecule called Reticulocalbin 1 (RCN1) and that RCN1 blocks cell stress and cell death, thereby allowing cells to survive and proliferate. Importantly, these findings will allow for the generation of drugs that block the function of EGFRvIII and RCN1 with the hope that these drugs will induce brain cancer cell death.

**Abstract:**

Reticulocalbin 1 (RCN1) is an endoplasmic reticulum (ER)-residing protein, involved in promoting cell survival during pathophysiological conditions that lead to ER stress. However, the key upstream receptor tyrosine kinase that regulates RCN1 expression and its potential role in cell survival in the glioblastoma setting have not been determined. Here, we demonstrate that RCN1 expression significantly correlates with poor glioblastoma patient survival. We also demonstrate that glioblastoma cells with expression of EGFRvIII receptor also have high RCN1 expression. Over-expression of wildtype EGFR also correlated with high RCN1 expression, suggesting that EGFR and EGFRvIII regulate RCN1 expression. Importantly, cells that expressed EGFRvIII and subsequently showed high RCN1 expression displayed greater cell viability under ER stress compared to EGFRvIII negative glioblastoma cells. Consistently, we also demonstrated that RCN1 knockdown reduced cell viability and exogenous introduction of RCN1 enhanced cell viability following induction of ER stress. Mechanistically, we demonstrate that the EGFRvIII-RCN1-driven increase in cell survival is due to the inactivation of the ER stress markers ATF4 and ATF6, maintained expression of the anti-apoptotic protein Bcl-2 and reduced activity of caspase 3/7. Our current findings identify that EGFRvIII regulates RCN1 expression and that this novel association promotes cell survival in glioblastoma cells during ER stress.

## 1. Introduction

Glioblastoma is the most aggressive and lethal brain tumor in adults mainly due to its highly proliferative, highly invasive nature and pro-survival features [1,2,3]. These tumorigenic characteristics require a high demand for intracellular protein synthesis and folding, and high and continuous levels of oxygen and nutrients such as glucose within the tumor microenvironment [4]. The endoplasmic reticulum (ER) is responsible for the correct folding of secretory and membrane bound proteins in mammalian cells [4,5]. Under conditions of high protein synthesis and folding, low oxygen or nutrient depletion commonly seen in glioblastoma cells and its microenvironment, the ER is unable to manage appropriate protein folding, resulting in the induction of ER stress [4,6]. The unfolded protein response (UPR) is a collection of adaptive signaling pathways that sense ER stress and either promote cell survival, or, when ER stress is too severe or prolonged, trigger apoptosis [7,8]. When the UPR is activated, the glucose-regulated protein 78 (GRP78) unbinds three protein sensors in the ER: inositol requiring enzyme 1 (IRE1), double-stranded RNA-activated protein kinase (PKR)-like ER kinase (PERK) which leads to increased expression of activating transcription factor 4 (ATF4), and activating transcription factor 6 (ATF6) that trigger parallel signaling pathways [9,10,11]. Activation of these pathways results in the restoration of protein homeostasis through the inhibition of new protein synthesis; the promotion of protein folding; the increase in ER-associated protein degradation and increasing the quantity of chaperones in the ER [12,13]. However, if ER stress cannot be resolved, the UPR switches from an adaptive survival mode towards the induction of apoptosis, often by modulating the expression of pro- and anti-apoptotic proteins including members of the Bcl-2 family and by increasing pro-apoptotic mechanisms such as the initiation of caspase activity [14,15,16,17].

Recently, a novel protein, Reticulocalbin 1 (RCN1) which belongs to the CREC (Calumenin, Reticulocalbin 1 and 3, ERC-55, Cab-45) family was found to suppress ER stress-mediated apoptosis in murine melanoma and renal cancer cells [5]. The RCN1 gene is located at the 11p13 chromosome and encodes for a highly conserved calcium-binding protein. RCN1 contains six calcium-binding motifs, a HDEL sequence and an ER retention signal [18]. RCN1 has been reported in several malignancies [18,19,20,21], however, how its expression is modulated and what precise role RCN1 plays in promoting tumorigenesis is not completely understood. Currently, there is no information about any functional role of RCN1 in glioblastoma progression. Amplification of the *EGFR* gene and subsequent over-expression of EGFR protein is a common genetic alteration in primary glioblastoma, with a frequency of approximately 40% [22,23,24]. The EGFR is activated upon ligand binding leading to subsequent downstream signaling cascades that mediate increased proliferation, migration, invasion and survival [25]. Over-expression of the EGFR in glioblastoma is often accompanied by rearrangements of the EGFR gene leading to the expression of EGFR variants [26,27]. The most common variant is the EGFRvIII, which is not expressed on normal non-tumorigenic tissue [26,28,29]. EGFRvIII is a mutated EGFR generated by a deletion between exons 2–7 producing a truncated receptor that lacks 267 amino acids in the extracellular binding domain [30,31]. This deletion is thought to generate a conformational change in the intracellular domains that permits the receptor to be constitutively active without ligand binding [32,33]. EGFRvIII is found in about 60% of EGFR-amplified glioblastomas. Additionally, in vivo models have shown that EGFRvIII-expressing tumors are more highly tumorigenic than wild-type EGFR-expressing tumors [34]. The EGFRvIII also provides increased survival following chemotherapy and radiotherapy potentially by modulating the expression and activation of apoptosis regulating proteins [35,36]. However, the role of the EGFRvIII in regulating survival/apoptosis in response to ER stress remains unknown. In this study, we investigate the possible relationship between the EGFR, RCN1 and cell survival under ER stress conditions.

## 2. Results

### 2.1. RCN1 Expression Correlates with Glioma Grade and Glioblastoma Patient Survival

**Figure 1:**
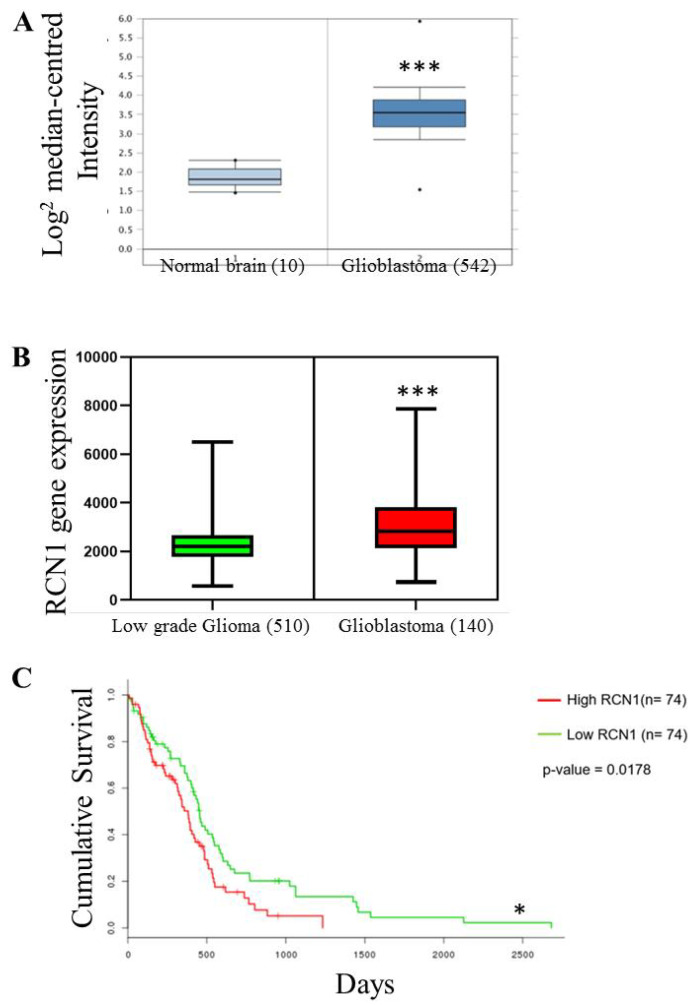
RCN1 expression correlates with glioma grade and poorer survival in glioblastoma patients. (**A**) Data set obtained from Oncomine (https://www.oncomine.org, access date 26 April 2018) showed that RCN1 gene expression is 3.22 times higher in glioblastoma patient tumor tissue (*n* = 542) compared to normal brain tissue (*n* = 10). The y-axis indicates the log2 median mRNA levels. (**B**) Box-plot comparison analysis performed with TCGA data available in OncoLnc (http://www.oncolnc.org/, access date 26 April 2018) at 50:50 upper and lower percentile, and indicated reduced RCN1 mRNA levels in low grade glioma (*n* = 510) compared to glioblastoma (*n* = 140). (**C**) The relationship between high (Red) and low (Green) RCN1 gene expression with patient survival was determined through mining a SurvExpress TCGA dataset. Kaplan–Meier survival curves were evaluated from the TCGA, *n* = 148. * *p* < 0.05; *** *p* < 0.001.

As RCN1 has recently been shown to correlate with poorer survival in prostate and lung cancer patients [37,38] we firstly examined its expression profiles in glioma patient samples using the TCGA database. Oncomine data mining using the TCGA dataset (*n* = 542; IDH wt and IDH mutant samples) revealed that RCN1 expression was significantly higher in glioblastoma tissue compared to normal brain tissue (Figure 1A). Similarly, available TCGA data from OncoLnc consisting of 650 patients illustrated that RCN1 expression was higher in glioblastoma compared to low grade glioma (Figure 1B). Importantly, TCGA data taken from SurvExpress (*n* = 148) also revealed that glioblastoma patients with tumors that contained higher RCN1 expression had significantly poorer overall survival compared to patients with glioblastoma tissue with lower RCN1 expression (Figure 1C).

### 2.2. RCN1 Is Highly Expressed in EGFRvIII Positive Glioblastoma Cells

**Figure 2:**
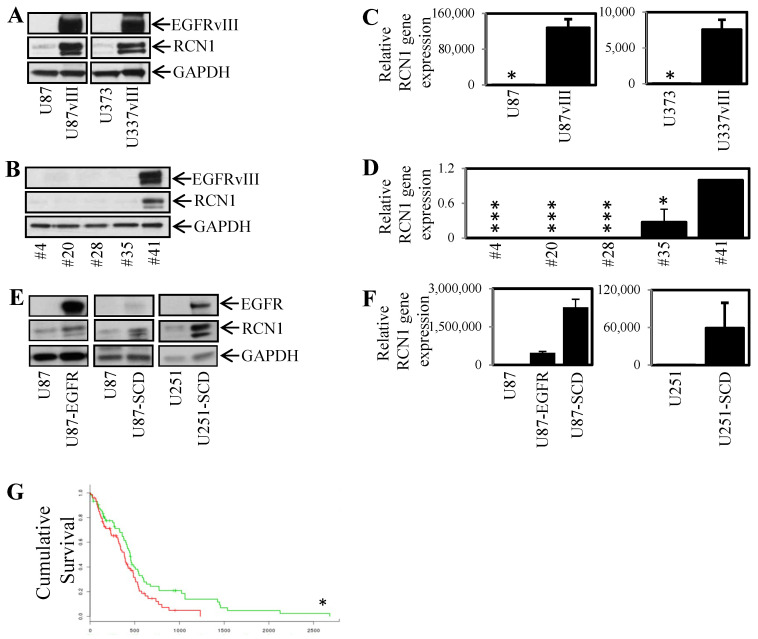
EGFRvIII expression correlates with RCN1 expression in glioblastoma cell lines. (**A**,**B**). A series of EGFRvIII positive and negative glioblastoma cell lines were lysed and assessed for EGFRvIII, RCN1 and GAPDH expression by Western blot. (**C**,**D**) The same cell lines as above were assessed for RCN1 gene expression by qPCR. Three cell lines with variant sub-populations with differing EGFR expression were (**E**) lysed and assessed for EGFR, RCN1 and GAPDH expression by Western blot and (**F**) RCN1 gene expression by qPCR. (**G**) The relationship between high (Red) and low (Green) EGFR and RCN1 gene expression with patient survival was determined through mining a SurvExpress TCGA dataset. Kaplan–Meier survival curves were evaluated from the TCGA, *n* = 148. * *p* < 0.05; *** *p* < 0.001.

Given that RCN1 correlated with glioblastoma patient survival, we next examined the expression of RCN1 in a series of commercially available and patient-derived primary glioblastoma cell lines established at the Royal Melbourne Hospital. Interestingly, RCN1 protein expression was substantially greater in cells stably transfected with EGFRvIII (U87vIII and U373vIII) compared to their parental EGFRvIII negative counterparts (U87 and U373; Figure 2A). Likewise, RCN1 expression was greatest in the primary glioblastoma cell line #41, which naturally expresses the EGFRvIII compared to 4 other primary glioblastoma cell lines that do not express EGFRvIII (#4, #20, #28 and #35; Figure 2B). Consistently, gene expression of RCN1 was significantly higher in EGFRvIII positive cells compared to the EGFRvIII negative expressing cells (Figure 2C,D). We have isolated a sub-population of cells originally from the #41 cell line—designated #41-SCD for single cell dilution. This sub-clone displays undetectable levels of EGFRvIII expression and, similarly to the above results, displayed significantly less RCN1 expression compared to the #41 parental cell line (Figure S1). Therefore, EGFRvIII expression correlated with RCN1 expression in glioblastoma cell lines. However, immunoprecipitation assays showed that EGFRvIII did not associate with RCN1 (Figure S2). Interestingly, a wt EGFR/RCN1 association was observed in both U87vIII and U373vIII RCN1 immunoprecipitated proteins (Figure S2).

As EGFRvIII expression correlated with RCN1 expression and as the wt EGFR associated with RCN1, we next examined 3 glioblastoma cell lines with increased wt EGFR compared to their parental counterparts either through stable transfection (U87wtEGFR) or through selection and re-population of single cells isolated from the original overall parental population—designated U87-SCD and U251-SCD for single cell dilution. The U87-wtEGFR, U87-SCD and the U251-SCD which all express greater wt EGFR than their parental counterparts all expressed greater levels of RCN1 both at the protein (Figure 2E) and gene expression level (Figure 2F). In addition, TCGA data taken from SurvExpress (*n* = 148; IDH wt and IDH mutant samples) indicated that patients with high levels of both RCN1 and EGFR had reduced survival time compared to patients with low expression of both genes (Figure 2G). Analysis of TCGA data assessing whether patients with high EGFRvIII and RCN1 expression could not be performed due to EGFRvIII data not currently present in these publicly assessable databases.

Surprisingly, the regulation of RCN1 expression by the wt EGFR and EGFRvIII did not require receptor kinase activity but only the presence of either the wt EGFR or EGFRvIII, suggesting a possible undiscovered kinase independent pro-tumorigenic function for EGFRvIII. EGF stimulation of U87wtEGFR cells did not increase RCN1 expression compared to unstimulated cells (Figure S3A) while the U87 cell line with the dead kinase version of the EGFRvIII (U87-DK) expressed similar levels of RCN1 than that of the U87vIII cell line (Figure S3B) despite having undetectable levels of phosphorylated EGFRvIII. Similarly, the treatment of the naturally EGFRvIII-expressing cell line #41 with the EGFR tyrosine kinase inhibitors erlotinib or gefitinib successfully reduced the phosphorylation of EGFRvIII but did not reduce the expression of RCN1 (Figure S3C). Taken together this data indicates that EGFR and EGFRvIII expression regulates RCN1 expression independently of their kinase activity.

### 2.3. RCN1 Expression Correlates with Increased Survival after the Initiation of ER Stress

**Figure 3:**
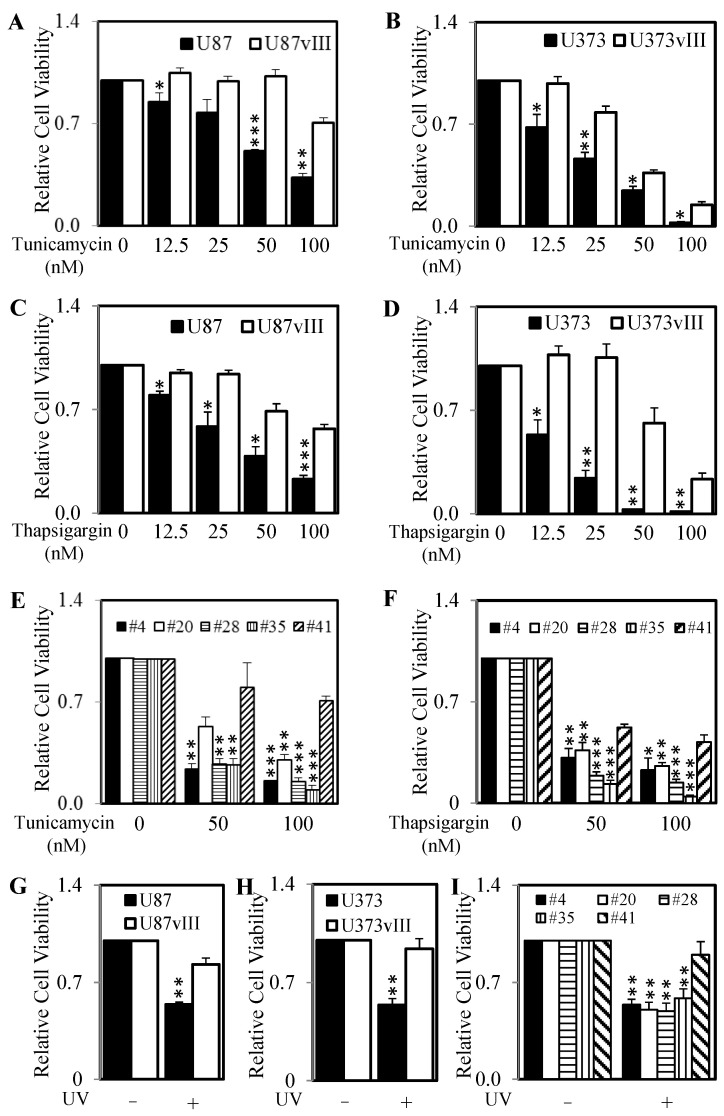
EGFRvIII expression correlates with increased survival after exposure to tunicamycin, thapsigargin and UV light. (**A**–**D**) U87, U87vIII, U373 and U373vIII cells were treated with increasing doses of tunicamycin (**A**,**B**) or thapsigargin (**C**,**D**) for 72 h. Cell viability was then determined using a commercially available Cell Titer-Glo kit and samples read on a bioluminometer. Data are expressed as % viability compared to untreated cells ± S.D of at least 3 independent experiments, each with 3 experimental replicates. #4, #20, #28, #35 and #41 glioblastoma cells were treated with increasing doses of (**E**) tunicamycin or (**F**) thapsigargin for 72 h. Cell viability was then determined as described above. (**G**) U87, U87vIII, (**H**) U373, U373vIII and (**I**) #4, #20, #28, #35 and #41 glioblastoma cells were exposed to UV light for 10 min and cell viability was examined following another 72 h as determined above. * *p* < 0.05; ** *p* < 0.01; *** *p* < 0.001.

RCN1 has recently been shown to play a role in ER stress [5]. Therefore, we next examined the cell viability of high and low RCN1 expressing cells following the pharmacological initiation of ER stress by tunicamycin and thapsigargin. Both, the U87vIII and U373vIII cell lines (high RCN1 expressing cells) displayed significantly greater cell survival compared to their parental controls (low RCN1 expressing cells) after treatment with increasing doses of tunicamycin (Figure 3A,B) and thapsigargin (Figure 3C,D). Similarly, the primary glioblastoma cell line #41 (high RCN1 expressing cell) displayed significantly greater cell survival compared to the 4 low RCN1 expressing glioblastoma cell lines (#4, #20, #28 and #35) after treatment with tunicamycin (Figure 3E) and thapsigargin (Figure 3F). To confirm these results, we assessed cell survival following the exposure of cells to UV light—another ER stress inducer. Consistently, all high RCN1 expressing cells (U87vIII, U373vIII and #41) displayed significantly greater cell survival compared to their low RCN1 expressing counterparts (U87, U373, #4, #20, #28 and #35) after being exposed to UV light (Figure 3G–I).

In addition, the survival of U87wtEGFR, U87-DK, U87-SCD and U251-SCD cells (which all have higher levels of RCN1 expression compared to their parental counterparts) was also significantly greater compared to the survival of control cells after treatment with tunicamycin (Figure S4A,B), thapsigargin (Figure S4C,D) or UV exposure (Figure S4E,F). Finally, as glucose deprivation and the commonly used glioblastoma patient chemotherapeutic, Temozolomide also induces ER stress we determined the survival of our RCN1 expressing cells in glucose-free media and after temozolomide treatment. Both, the U87vIII and U373vIII cell lines (high RCN1 expressing cells) displayed significantly greater cell survival compared to their parental controls (low RCN1 expressing cells) after being cultured in media free of glucose or after being challenged with temozolomide (Figure S4G,H).

### 2.4. Knockdown of RCN1 Significantly Decreases Cell Survival and RCN1 Over-Expression Significantly Increases Cell Survival Following the Initiation of ER Stress

**Figure 4:**
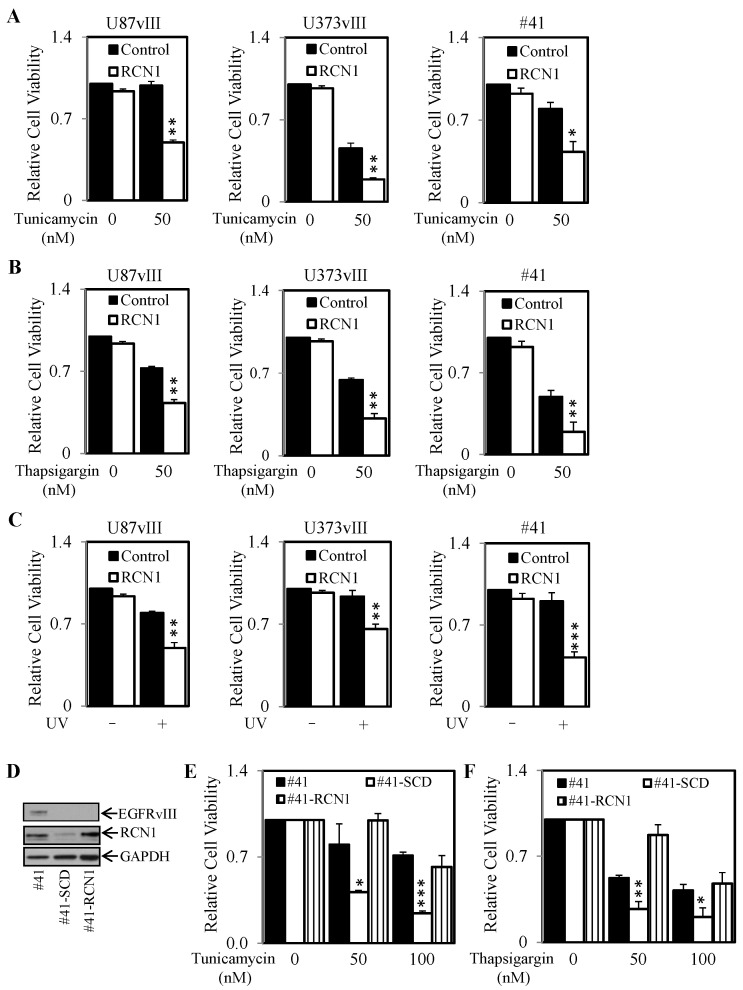
RCN1 knockdown reduces and RCN1 over-expression increases cell survival after exposure to tunicamycin, thapsigargin and UV light. U87vIII, U373vIII and #41 glioblastoma cells were transfected with control (■) or RCN1 (□) siRNA and then treated with (**A**) ± tunicamycin (50 nM), (**B**) ± thapsigargin (50 nM), or (**C**) ± exposure to UV light. Cell viability was then determined after 72 h using a commercially available Cell Titer-Glo kit and samples read on a bioluminometer as described in Figure 3. Data are expressed as % viability compared to untreated cells ± S.D of at least 3 independent experiments, each with 3 experimental replicates. (**D**) #41, #41-SCD and #41-RCN1 cells were lysed and assessed for EGFRvIII, RCN1 and GAPDH expression by Western blot. #41 (■), #41-SCD (□) and #41-RCN1 (bars with vertical lines) cells were treated with (**E**) ± tunicamycin (50 nM) or (**F**) ± thapsigargin (50 nM) and cell viability was then determined after 72 h as described above. * *p* < 0.05; ** *p* < 0.01; *** *p* < 0.001.

As EGFRvIII and RCN1 expression correlated with the increased ability of cells to survive treatment of ER stress inducers, we next knocked down RCN1 expression using siRNA and determined the effect of this knockdown when challenged with tunicamycin, thapsigargin or UV exposure. Following knockdown of RCN1, the 3 EGFRvIII expressing cells: U87vIII, U373vIII and #41 all displayed reduced cell survival when treated with tunicamycin (Figure 4A), thapsigargin (Figure 4B) and UV exposure (Figure 4C) compared to cells transfected with control siRNA. As low RCN1 expression levels or knockdown of RCN1 correlated with reduced cell survival following the initiation of ER stress (tunicamycin or thapsigargin treatment), we next determined if over-expression of RCN1 via stable transfection could promote cell survival following tunicamycin or thapsigargin treatment. To perform these experiments, the #41-SCD cell line (low RCN-1 levels) was stably transfected with an RCN1 construct to yield a cell line (#41-RCN1) that expresses similar levels to the original #41 cell line (Figure 4D). Importantly, this re-introduction of RCN1 led to these cells displaying increased survival compared to the #41-SCD cell line after tunicamycin or thapsigargin treatment (Figure 4E,F). This level of survival was comparable to that seen in the original high RCN1 expressing #41 cell line.

### 2.5. RCN1 Expression Reduces ATF6 Activity When Challenged with Tunicamycin and Thapsigargin

Tunicamycin and thapsigargin initiate ER stress through the activation of 3 pathways (PERK-ATF4, IRE1-XBP1 and ATF6) collectively called the unfolded protein response. This response triggers apoptosis in cells if the ER stress is too severe or prolonged.

**Figure 5:**
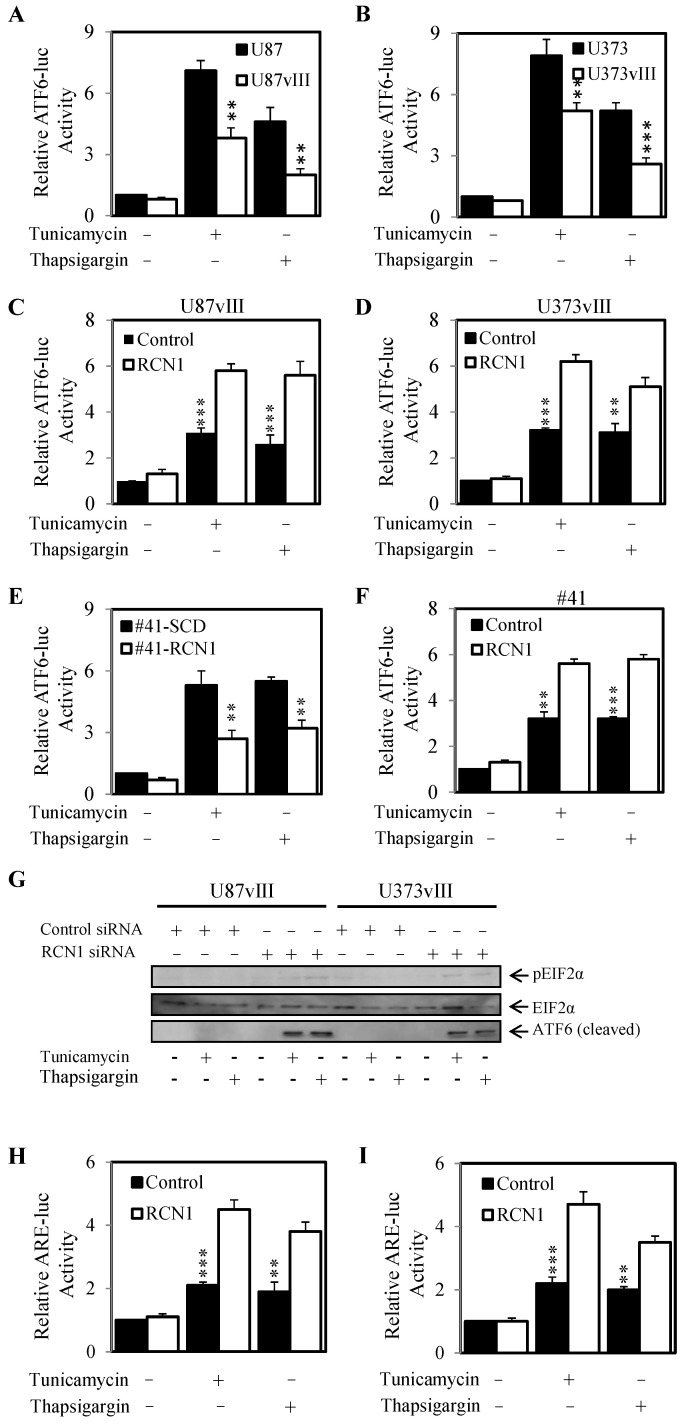
RCN1 expression inhibits Er stress response. (**A**) U87 and U87vIII and (**B**) U373 and U373vIII cells were transfected with the ATF6 luciferase reporter construct and allowed to adhere overnight. Cells were then treated with ± tunicamycin (50 nM) or ± thapsigargin (50 nM) for a further 24 h, lysed and assessed for luciferase activity. Data are expressed as relative luciferase activity (fold change) by standardizing the luciferase activity of the control untreated cells to 1, and accordingly normalizing all other raw values. (**C**) U87vIII and (**D**) U373vIII cells were transfected with either control (■) or RCN1 (□) siRNA and the ATF6 luciferase reporter construct and allowed to adhere overnight. Cells were then treated with ± tunicamycin or ± thapsigargin for a further 24 h, lysed and assessed for luciferase activity. Data are expressed as relative luciferase activity (fold change) by standardizing the luciferase activity of the control siRNA, control treated cells to 1, and accordingly normalizing all other raw values. (**E**) #41-SCD and #41-RCN1 cells were transfected with the ATF6 luciferase reporter construct and allowed to adhere overnight. Cells were then treated with ± tunicamycin or ± thapsigargin for a further 24 h, lysed and assessed for luciferase activity as outlined above. (**F**) #41 cells were transfected with either control or RCN1 siRNA and the ATF6 luciferase reporter construct and allowed to adhere overnight. Cells were then treated with ± tunicamycin (50 nM) or ± thapsigargin (50 nM) for a further 24 h, lysed and assessed for luciferase activity as outlined above. (**G**) U87vIII and U373vIII cells were transfected with control or RCN1 siRNA and then treated with ± tunicamycin (50 nM) or ± thapsigargin (50 nM) for a further 24 h, lysed and assessed for pEIF2α, total EIF2α and ATF6 expression by Western blot. (**H**) U87vIII and (**I**) U373vIII cells were transfected with either control or RCN1 siRNA and the ARE (antioxidant response element) luciferase reporter construct and allowed to adhere overnight. Cells were then treated with ± tunicamycin (50 nM) or ± thapsigargin (50 nM) for a further 24 h, lysed and assessed for luciferase activity as outlined above. ** *p* < 0.01; *** *p* < 0.001.

As we previously demonstrated that EGFRvIII and RCN1 expression correlated with enhanced cell survival under ER stress conditions, we next evaluated whether ATF6 activity varied in high or low expressing RCN1 glioblastoma cells. Indeed, the U87vIII and U373vIII cell lines (high RCN1 expressing cells) displayed significantly lower ATF6 activity compared to their parental controls (low RCN1 expressing cells) after treatment with tunicamycin and thapsigargin (Figure 5A,B). Knockdown of RCN1 in these cells resulted in an elevated level of ATF6 activity following treatment with tunicamycin and thapsigargin (Figure 5C,D). In addition, the exogenous RCN1 over-expressing cell line (#41-RCN1) displayed significantly less ATF6 activity compared to the un-transfected control cells (#41-SCD) when treated with tunicamycin or thapsigargin (Figure 5E). Conversely, tunicamycin and thapsigargin treatment caused a significant increase in ATF6 activity in #41 cells with knocked down RCN1 expression compared to the ATF6 activity levels in #41 cells transfected with control siRNA (Figure 5F).

As we observed that RCN1 expression correlated with ATF6 activity, we next determined if its expression also regulated other members of the unfolded protein response pathways. Indeed, knockdown of RCN1 resulted in increased phosphorylation of EIF2α in both U87vIII and U373vIII cell lines when treated with tunicamycin and thapsigargin (Figure 5G). Knockdown of RCN1 also resulted in an increase in expression of the cleaved ATF6 (which is involved in the ER stress response) when treated with tunicamycin and thapsigargin and thus these results were consistent with our ATF6 luciferase activity data (Figure 5G). Finally, we assessed changes in the antioxidant response element (a surrogate marker for NRF2 activity) and increases in ATF4 expression which are both regulated by PERK phosphorylation. We demonstrated that knockdown of RCN1 led to increases in ATF4 expression (Figure 5G) and increases in anti-oxidant response (NRF2 activity) when cells were challenged with tunicamycin and thapsigargin (Figure 5H,I). Taken together this data indicates that EGFRvIII expression and subsequent RCN1 expression inhibits UPR activity when challenged with ER stress inducers tunicamycin and thapsigargin.

### 2.6. RCN1 Expression Protects Cells from ER Stress-Mediated Apoptosis

**Figure 6:**
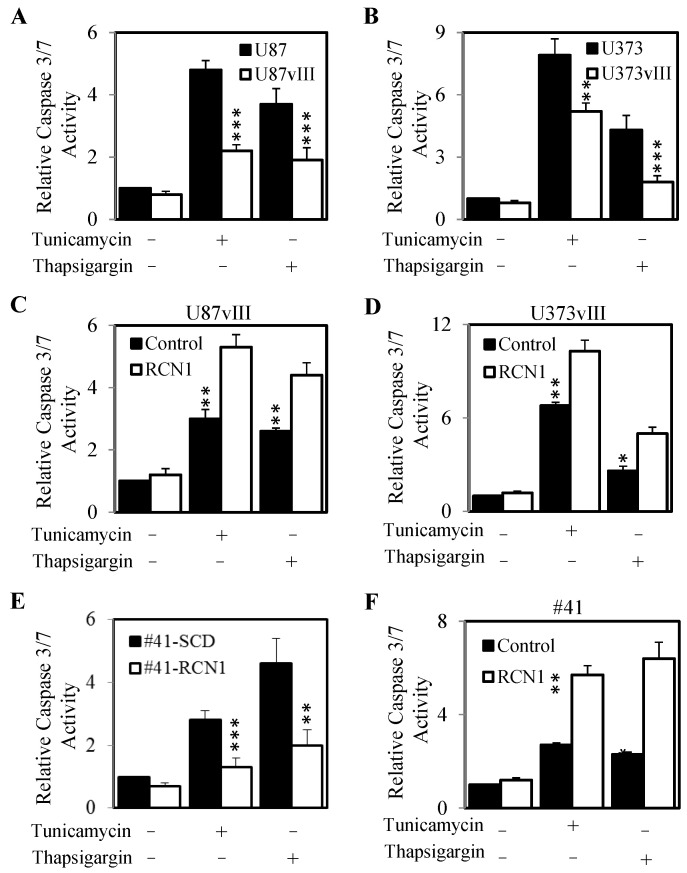
RCN1 expression inhibits caspase 3/7 activity. (**A**) U87 and U87vIII and (**B**) U373 and U373vIII cells were seeded and allowed to adhere overnight. Cells were then treated with ± tunicamycin (50 nM) or ± thapsigargin (50 nM) for a further 24 h, lysed and assessed for caspase 3/7 activity. Data are expressed as relative caspase 3/7 activity (fold change) by standardizing the caspase 3/7 activity of the control untreated cells to 1, and accordingly normalizing all other raw values. (**C**) U87vIII and (**D**) U373vIII cells were transfected with either control (■) or RCN1 (□) siRNA and allowed to adhere overnight. Cells were then treated with ± tunicamycin or ± thapsigargin for a further 24 h, lysed and assessed and assessed for caspase 3/7 activity. Data are expressed as relative caspase 3/7 activity (fold change) by standardizing the caspase 3/7 activity of the control siRNA, control treated cells to 1, and accordingly normalizing all other raw values. (**E**) #41-SCD and #41-RCN1 cells were seeded and allowed to adhere overnight. Cells were then treated with ± tunicamycin or ± thapsigargin for a further 24 h, lysed and assessed for caspase 3/7 activity as outlined above. (**F**) #41 cells were transfected with either control or RCN1 siRNA allowed to adhere overnight. Cells were then treated with ± tunicamycin (50 nM) or ± thapsigargin (50 nM) for a further 24 h, lysed and assessed for caspase 3/7 activity as outlined above. * *p* < 0.05; ** *p* < 0.01; *** *p* < 0.001.

As our EGFRvIII and high RCN1 expressing cells displayed greater survival after treatment with tunicamycin and thapsigargin, we next evaluated whether this was due to a reduction in the initiation of apoptosis. Indeed, the U87vIII and U373vIII cell lines (high RCN1 expressing cells) displayed significantly lower caspase 3/7 activity compared to their parental controls (low RCN1 expressing cells) after treatment with tunicamycin or thapsigargin (Figure 6A,B). Knockdown of RCN1 in these cells resulted in an elevated level of caspase3/7 activity following treatment with tunicamycin and thapsigargin (Figure 6C,D). In addition, the exogenous RCN1 over-expressing cell line (#41-RCN1) displayed significantly less caspase 3/7 activity compared to the un-transfected control cells (#41-SCD) when treated with tunicamycin or thapsigargin (Figure 6E). Conversely, tunicamycin and thapsigargin treatment caused a significant increase in caspase 3/7 activity in #41 cells with knocked down RCN1 expression compared to the caspase 3/7 activity levels in #41 cells transfected with control siRNA (Figure 6F).

**Figure 7:**
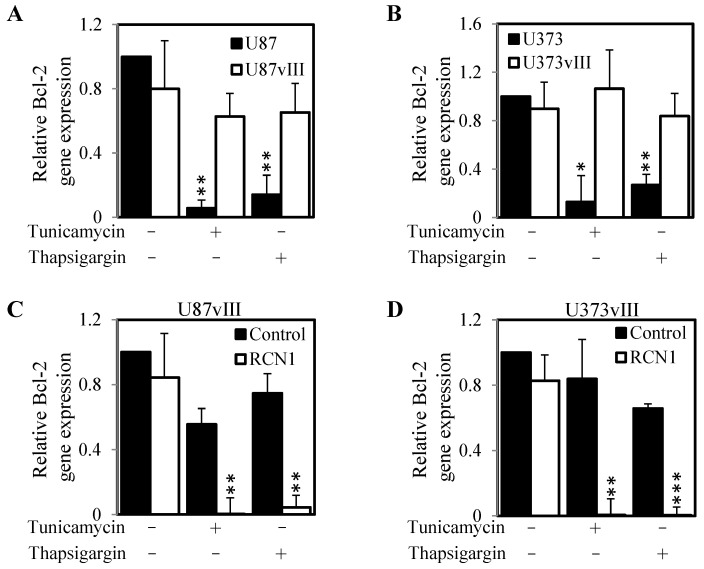
RCN1 expression prevents reduction in Bcl-2 gene expression. (**A**) U87 and U87vIII and (**B**) U373 and U373vIII cells were seeded and allowed to adhere overnight. Cells were then treated with ± tunicamycin (50 nM) or ± thapsigargin (50 nM) for a further 24 h, then lysed and assessed for Bcl-2 gene expression by qPCR. Similarly, (**C**) U87vIII and (**D**) U373vIII cells were transfected with either control or RCN1 siRNA and allowed to adhere overnight. Cells were then treated with ± tunicamycin (50 nM) or ± thapsigargin (50 nM) for a further 24 h, lysed and assessed for Bcl-2 gene expression by qPCR luciferase activity. Data are expressed as relative Bcl-2 gene expression (fold change) by standardizing the Bcl-2 gene expression of the control untreated cells to 1, and accordingly normalizing all other raw values. * *p* < 0.05; ** *p* < 0.01; *** *p* < 0.001.

We next evaluated whether the differences in caspase 3/7 activity reflected similar differences in the gene expression of the anti-apoptotic molecule Bcl-2. Consistently, treatment with tunicamycin or thapsigargin led to significant reduction in Bcl-2 gene expression in both EGFRvIII negative parental cell lines U87MG and U373MG (low RCN1 expressing cells). However, this tunicamycin or thapsigargin induced reduction of Bcl-2 gene expression was not seen in the U87vIII and U373vIII (high RCN1 expressing cells) (Figure 7A,B). However, knockdown of RCN1 in U87vIII and U373vIII cells resulted in a significantly reduced level of Bcl-2 gene expression following treatment with tunicamycin and thapsigargin compared to cells transfected with control siRNA (Figure 7C,D). Taken together this data indicates that EGFRvIII and subsequent RCN1 expression inhibits cell apoptosis when challenged with ER stress inducers tunicamycin and thapsigargin by reducing caspase 3/7 activity and maintaining the expression of the anti-apoptotic protein Bcl-2.

## 3. Discussion

Previous findings have demonstrated that the expression of the ER-localized, calcium binding protein RCN1 correlates with progression of breast, liver, kidney, lung, prostate and colorectal cancer [19,20,21,37,38,39] and may play an important role in mediating resistance to treatment in uterine and nasopharyngeal carcinoma [40]. However, it is unclear as to which receptor tyrosine kinases regulate RCN1 expression and whether RCN1 plays a role in glioblastoma progression. Our data begins to address these unanswered important questions initially evaluating online databases. Consistently with the above studies, our data mining demonstrated that high RCN1 expression correlates with increasing glioma grade and predicts poorer survival in glioblastoma patients suggesting a potential role of RCN1 in promoting glioblastoma progression. To begin to elucidate this potential pro-tumorigenic role, we determined the gene and protein expression of RCN1 in several commercially available parental cell lines, single cell variant sub-populations, stably transfected and patient-derived primary and recurrent glioblastoma cell lines. We discovered that the expression of transfected or naturally occurring EGFRvIII and wildtype EGFR strongly correlates with increased RCN1 expression suggesting that the EGFR and EGFRvIII can regulate RCN1 expression.

In addition, we provide several lines of evidence that the kinase activity of the EGFR or EGFRvIII is not required for this enhanced RCN1 expression. Firstly, EGF stimulation of wt EGFR leading to enhanced EGFR phosphorylation did not enhance RCN1 expression. Secondly, the expression of a dead kinase EGFRvIII in U87MG cells (U87-DK) displayed comparable RCN1 expression to that of the U87vIII (with functional EGFRvIII kinase) and significantly greater RCN1 expression to the parental U87MG (EGFRvIII negative) cell line. Finally, Gefitinib and Erlotinib could successfully reduce EGFRvIII phosphorylation but had no effect on RCN1 expression in EGFRvIII expressing cells. This data indicates that the positive correlation between EGFRvIII and RCN1 expression is independent of the EGFRvIII kinase activity. Although the full-length wt EGFR has been previously shown to have several oncogenic features that does not require its kinase activity [41,42,43], to the best of our knowledge our study is to first to identify a kinase-independent property of the EGFRvIII. How EGFRvIII correlates with RCN1 expression without the requirement for kinase activity is unknown. We can only speculate that the EGFRvIII expression has been identified in the mitochondria [44,45] and that this localization may in turn lead to changes in localization, expression or activation of key survival proteins/pathways that regulate ER stress and apoptosis. Likewise, the EGFRvIII has been identified in the nucleus [46,47]. Whether the EGFRvIII can induce gene transcription of pro-survival markers such as RCN1 without kinase activity is not well known. Nonetheless, we identify that EGFRvIII expression correlates to RCN1 expression. However, despite correlative expression of EGFRvIII and RCN1, our immunoprecipitation studies demonstrated that EGFRvIII and RCN1 did not associate or immuno-complex with each other. Similar to Oldrini et al. who reported that RCN1 binds EGF-stimulated wt EGFR in the epidermoid carcinoma cell line A431 [48], we found that RCN1 could associate with the wt EGFR in both U87vIII and U373vIII cells despite these cell lines expressing greater levels of EGFRvIII than wt EGFR. This disparity therefore is not due to total assessable protein levels but potentially due to the conformational change in the EGFRvIII.

The EGFRvIII variant receptor has previously been shown to promote enhanced survival in glioblastoma cells through mechanisms involving the inhibition of apoptosis. Our current data supports this notion and provides the first line of direct evidence that the EGFRvIII can suppress apoptosis when cells are under ER stress. Specifically, we demonstrated that expression of the anti-apoptotic protein Bcl-2 and caspase 3/7 activity was significantly reduced in our EGFRvIII expressing cell lines compared to parental cell lines when challenged with ER stress inducers tunicamycin and thapsigargin. Similarly, Nagane and colleagues [36] showed that U87vIII cells expressed significantly greater expression of the anti-apoptotic protein Bcl-X_L_ and reduced caspase-3-like protease activity compared to U87vIII cells when challenged with cisplatin; a chemotherapeutic that has been shown to induce ER stress-mediated apoptosis [49,50]. Interestingly, these differences in apoptotic markers between EGFRvIII negative and positive expressing cells were not seen in optimal growth conditions in vitro but only emerge in sub-optimal micro-environments often encountered by glioblastoma cells in patients including drug challenged [36], hypoxic environments [51], in vivo growth [34], and when cultured in media without serum [34]; all inducers of ER stress.

Similarly, RCN1 has been shown to play a pro-survival role during ER stress. Several reports indicating that RCN1 is responsible for promoting tumor survival in prostate, liver, kidney and skin cancer cell lines under ER stress [5,38]. In addition, RCN1 depletion induced ER stress in animal models and led to cell death [38,40]. However, no study to date has evaluated whether EGFRvIII and RCN1 co-operate to promote glioblastoma survival. Our current data showed that cells that were positive for EGFRvIII expression and subsequently expressed high RCN1 levels displayed significantly greater cell survival when challenged with pharmacological and environmental ER stress inducers: tunicamycin, thapsigargin, UV exposure or reduced glucose concentration. Importantly, exogenous introduction of RCN1 into cells also led to enhanced survival of cells when ER stress was induced, while knockdown of RCN1 reversed this enhanced survival in ER stress conditions. These data support the notion that EGFRvIII provides a pro-survival, RCN1-dependent mechanism in sub-optimal, ER stress conditions. Similarly, cells with transfected or sub-populations selected from single cell cultures displaying enhanced wt EGFR and subsequently higher levels of RCN1 also demonstrated greater survival when treated with tunicamycin, thapsigargin or exposed to UV irradiation. Our isolated variant U87 and U251 sub-populations contained higher EGFR expression compared to their parental population. This provides evidence that intra-tumor heterogeneity exists within cell lines, in which the genetic landscape differs within a particular cluster of cells. Therefore, these cells may exhibit varying resilience to sub-optimal conditions such as low nutrient conditions, hypoxia or drug treatment compared to neighboring cells in the same tumor population. This is particularly relevant in glioblastoma where cells utilize nutrients and oxygen quickly and are therefore often in sub-optimal microenvironments [52]. As such cells that can prevent ER stress-mediated apoptosis triggered by these sub-optimal conditions have a significant growth and survival advantage. We propose that sub-populations of cells with EGFRvIII, high wt EGFR and subsequent high RCN1 expression are likely to be more tolerant and better adapted to survive sub-optimal growth conditions often observed in the glioblastoma micro-environment. However, we propose this hypothesis with the caveat that most of our studies were performed with commercially available, high passage glioblastoma cells transfected with EGFRvIII (although we did also use a glioblastoma cell line that naturally expresses the EGFRvIII). This is a limitation of our study and many other EGFRvIII-based studies as the routine culturing of naturally expressing EGFRvIII cells is very rare.

Our current data where knockdown of RCN1 led to enhanced ATF6 activity and over-expression of RCN1 mediated reduced ATF6 activity in cells when challenged with ER stress inducers is consistent with other reports. Similar to our findings, Huang et al., showed that knockdown of RCN1 enhanced the expression of ER stress markers GRP78, CHOP, Herp, Erdj4, ATF4 and EDEM1 and promoted apoptosis in nasopharyngeal carcinoma cells when challenged with the chemotherapeutic adriamycin [40]. Our current data also showed that RCN1 knockdown leads to enhanced phosphorylation of EIF2α, increased expression of ATF4 and enhanced activity of NRF2, members of another UPR pathway PERK-EIF2α-ATF4 that is divergent to the ATF6 pathway. These results suggest that RCN1 may regulate more than one of the UPR pathways and as enhanced EIF2α phosphorylation and enhanced ATF4 expression after RCN1 knockdown has been reported in other cell types, this RCN1 regulation may be universal and not glioblastoma specific [5,38].

It has also been shown that over-expression of RCN1 led to reduced ER stress induction and reduced apoptosis when treated with adriamycin. Based on these findings we can speculate that the response to temozolomide and radiotherapy (the most used treatment strategy for glioblastoma patients’ post-surgery) may be dependent on whether this treatment can induce sustained ER stress and subsequent apoptosis and in turn this may be dependent on RCN1 expression. Indeed, our results showed that EGFRvIII expressing cells (with higher RCN1 expression) had greater survival when challenged with temozolomide compared to parental, EGFRvIII negative cell lines. Although, we did not expand these data to explore whether temozolomide enhanced UPR activity at varying levels based on RCN1 expression leading to the observed apoptosis, others have shown that temozolomide triggers enhanced UPR activity mediated cell death [53]. Furthermore, Dadey and colleagues showed that single dose radiation led to increased PERK expression and reduced cell viability in glioblastoma cells in vitro [54].

## 4. Materials and Methods

### 4.1. Antibodies and Reagents

The rabbit polyclonal antibodies directed against pEGFR, EGFR, pEIF2α, EIF2α and GAPDH and the mouse monoclonal antibody directed against ATF4 were all obtained from Cell Signaling Technology (Danvers, MA, USA). The RCN1 antibody was from Abcam (Cambridge, UK). The mouse monoclonal ATF6 antibody was from Enzo Life Sciences (Redfern, NSW, Australia). The EGFRvIII (LMH-144) antibody was generated in house at the Olivia Newton John Cancer Research Institute. Tunicamycin, thapsigargin and temozolomide were all purchased from Sigma (Sigma-Aldrich, St. Louis, MO, USA). EGF was purchased from Life Technologies (Life Technologies; Carlsbad, CA, USA) and the anti-EGFR inhibitors: Erlotinib and Gefitinib were obtained from Selleck Chemicals (Houston, TX, USA). The Luciferase Reporter Assay reagents were purchased from Promega (Madison, WI, USA). Human RCN1 and negative control siRNA were from ThermoFisher Scientific (Scoresby, VIC, Australia).

### 4.2. Cell Culture

The glioblastoma cell lines U87MG, U373MG and U251MG were purchased from ATCC. The stably transfected U87vIII, U87wtEGFR, U87-DK and U373vIII cell lines were all originally provided by Prof Webster Cavenee and Prof Frank Furnari (Ludwig Institute for Cancer Research, San Diego Branch, University of California at San Diego). The primary glioblastoma cell lines: #4, #20, #28, #35 and #41 were originally derived from 5 patients with pathologically confirmed glioblastoma at the Royal Melbourne Hospital and subsequently modified from neurosphere non-adherent cells to adherent cells grown in monolayer. Use of these cell lines in the laboratory was approved by the Melbourne Health Human Research and Ethics Committee (HREC 2012.219). All cells were maintained in Dulbecco’s Modified Eagle’s Medium (Life Technologies) contained 5% fetal bovine serum (FBS) (Life Technologies, Carlsbad, CA, USA), 100 U/mL penicillin and 100 µg/mL streptomycin (Life Technologies). Cells were incubated in a humidified atmosphere of 90% air and 10% CO_2_ at 37 °C. DMEM Glucose-free media was purchased from Life technologies.

### 4.3. Generation of Cells with Varying Levels of EGFR and EGFRvIII

U87MG, U251MG and #41 cells were seeded at an initial concentration of 1 × 10^3^ cells/mL and serially diluted 1:2 across a 96-well plate with DMEM to isolate one cell/well. These single cell clones were then allowed to proliferate, and expanded populations from single cell origin were analyzed for EGFR and EGFRvIII expression by Western blot and qPCR. U87MG and U251MG cells with increased EGFR expression compared to parental were designated U87-SCD and U251-SCD while #41 cells with decreased EGFRvIII expression compared to parental were designed #41-SCD.

### 4.4. Generation of Cells with RCN1 Over-Expression and siRNA Transfection

The #41-RCN1 transfected cell line was generated by transfecting cells with the pcDNA3.1-RCN-1 (Kindly provided by Prof Huiqing Yuan; Shandong University China; [38]) using FuGENE HD transfection reagent (Promega, Madison, WI, USA) following the manufacturer’s instructions and selected with Geneticin (Sigma-Aldrich, St. Louis, MO, USA). Confirmation of over-expression was performed by qPCR and Western blotting. Confirmed stably transfected cells (designated #41-RCN1) were maintained in standard cell culture conditions containing 1.0 mg/mL of Geneticin. For siRNA experiments cells were transiently transfected with RCN1 or control siRNA using the HiPerFect transfection reagent (Qiagen, Hilden, Germany) as per the manufacturer’s instructions for 24 h before subsequent treatments.

### 4.5. Cell Viability Assays

Cells were plated in 96-well plates and allowed to adhere overnight. Triplicate wells were treated with varying concentrations of tunicamycin, thapsigargin, exposed to UV (10 min) or cultured in glucose-free media for 3 days. Cells were then lysed and cell viability relative to an appropriate control was determined using a commercially available Cell Titer-Glo kit (Promega) following manufacturer’s instructions. Cell lysates were read on a bioluminometer.

### 4.6. Western Blotting and Immunoprecipitation

Cells were lysed in a lysis buffer (50 mM Tris (pH 7.4), 150 mM NaCl, 1% Triton-X-100, 50 mM NaF, 2 mM MgCl_2_, 1 mM Na_3_VO_4_ and protease inhibitor cocktail (Roche; Basel, Switzerland)) and clarified by centrifugation (13,000× *g* for 15 min at 4 °C). For immunoprecipitation assays, 500 µg of clarified lysates as determined by the Pierce BCA Protein Assay Kit (Thermo Scientific) was incubated with 5 µg of RCN1 or EGFRvIII (LMH-144) antibody and 30 µL of washed protein G Agarose affinity beads (Sigma) overnight at 4 °C. The next day immunoprecipitates were washed 3 times with lysis buffer and mixed with sample buffer (Life Technologies). Proteins were then separated by SDS-PAGE (Life Technologies), blotted onto nitrocellulose and probed with the indicated primary antibodies. The signal was visualized using an ECL chemiluminescence detection kit (GE Healthcare; Chicago, IL, USA) following incubation with appropriate secondary antibodies (Biorad Laboratories; Hercules, CA, USA).

### 4.7. RNA Extraction and RT-PCR

Cells were seeded in 6-well plates and allowed to adhere overnight. Following cell treatments and/or transfections, total RNA was extracted with the RNeasy Mini Kit (Qiagen; Hilden, Germany) following the manufacturer’s instructions. Reverse transcription was performed using the High Capacity RNA-to-cDNA Kit (Applied Biosystems; Waltham, MA, USA). Reverse Transcription-PCR was performed using the GeneAmp PCR System 2400 (Perkin Elmer, Waltham, MA) under the conditions of 37 °C for 60 min and 95 °C for 5 min at a reaction volume of 20 µL. In order to quantify the transcripts of the genes of interest, real-time PCR was performed using the ViiA 7 Real-Time PCR system (Applied Biosystems) for EGFR (Applied Biosystems, Hs01076090_m1), RCN1 (Hs01923804_g1), Bcl-2 (Applied Biosystems, Hs04986394_s1) and GAPDH (Applied Biosystems, Hs02758991_g1). The EGFRvIII specific primers were ordered using the sequences previously validated [55]. Amplified RNA samples was calculated using the 2^−∆∆CT^ method [56].

### 4.8. ATF6 and ARE-Luc Luciferase Assay

Cells were transfected with the ATF6 luciferase construct (pGL4.39; Promega) or the anti-oxidant response element luciferase construct (pGL4.37; Promega) with or without co-transfection with control and RCN1 siRNA and allowed to adhere overnight. After 24 h, cells were then treated with 0 and 50 nM of tunicamycin or thapsigargin for 24 h. Following another 24 h, cells were lysed and assessed for ATF6 luciferase activity with the use of the Luciferase Reporter Assay Kit (Promega) following the manufacturer’s instructions. Readings from lysed cells that were treated with contol (i.e., without inhibitors) were normalized to 1 and all subsequent readings were adjusted accordingly relative to control treated readings.

### 4.9. Caspase3/7 Assay

Cells were plated in 96-well plates and allowed to adhere overnight. Triplicate wells were treated with 0 and 50 nM of tunicamycin or thapsigargin for 24 h. Cells were then lysed and apoptosis was measured using the Caspase 3/7-Glo assay kit (Promega) following manufacturer’s instructions. Cell lysates were read on a bioluminometer.

### 4.10. OncoLnc (TCGA)

TCGA gene expression data was obtained from using the OncoLnc database (www.oncolnc.org), access date 26 April 2018. For a given gene, the gene ID was entered and ‘GBM’ was selected. Patients belonging to either the lower or upper 25th percentiles were chosen for the analysis.

### 4.11. Statistical Analysis

The statistical analyses for all Western blots, qRT-PCR and cell viability assays was conducted with an unpaired, two-tail Student’s *t*-test was used to test for significance and a minimum threshold of *p* < 0.05 was chosen to determine significance. The survival analyses from OncoLnc used a log-rank *t*-test to determine significance and data was displayed on a Kaplan–Meier plot.

## 5. Conclusions

In conclusion, our current findings have identified that the EGFR and EGFRvIII drive RCN1 gene and protein expression which protects glioblastoma cells from apoptosis when challenged with inducers of ER stress. This enhanced survival under ER stress conditions may be due to the inhibition of the activity of ATF6, EIF2α, NRF2 and caspase 3/7, the suppression of ATF4 expression and the maintained expression of the anti-apoptotic molecule Bcl-2. These findings that RCN1 expression confers increased survival in our cell lines and correlates with poorer glioblastoma patient survival suggest that targeting RCN1 therapeutically to promote sustained ER stress and subsequently, trigger apoptosis, may represent a promising alternative strategy for glioblastoma treatment.

## Data Availability

Data sharing is not applicable for this article.

## Supplementary Materials

The following are available online at https://www.mdpi.com/2072-6694/13/6/1198/s1, Figure S1: #41 and #41-SCD cells were lysed and assessed for EGFRvIII, RCN1 and GAPDH expression by western blot, Figure S2: U87vIII and U373vIII cells were lysed and proteins were immunoprecipitated using either the RCN1 or EGFRvIII antibody as described in materials and methods, Figure S3: RCN1 expression is not regulated by EGFR activity, Figure S4: U87, U87-EGFR, U87DK, U87-SCD, U251 and U251-SCD cells were treated with increasing doses of tunicamycin A, B or thapsigargin C, D for 72 h.
