# Supplementary material for: EGFRvIII Promotes Cell Survival during Endoplasmic Reticulum Stress through a Reticulocalbin 1-Dependent Mechanism
Source: Cancers (Basel). 2021 Mar 10;13(6):1198. doi: 10.3390/cancers13061198 (PMC7999088; doi:10.3390/cancers13061198)
Supplement: Supplementary file 1 [file cancers-13-01198-s001.pdf]

SupplementaryMaterials

EGFRvIII Promotes Cell Survival during Endoplasmic Reticulum Stress through a Reticulocalbin 1-Dependent Mechanism

Juliana Gomez, Zammam Areeb, Sarah F. Stuart, Hong P. T. Nguyen, Lucia Paradiso, Ahmad Zulkifli, Sonakshi Madan, Vijay Rajagopal, Magdalene K. Montgomery, Hui K. Gan, Andrew M. Scott, Jordan Jones, Andrew H. Kaye, Andrew P. Morokoff and Rodney B. Luwor

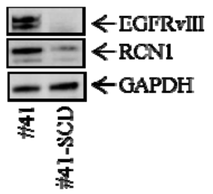

Figure S1. #41 and #41-SCD cells were lysed and assessed for EGFRvIII, RCN1 and GAPDH expression by western blot.

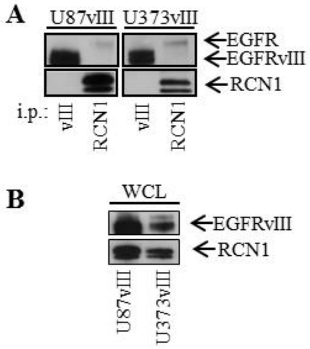

Figure S2. U87vIII and U373vIII cells were lysed and proteins were immunoprecipitated using either the RCN1 or EGFRvIII antibody as described in materials and methods. (**A**) Immunoprecipitated complexes were separated by gel electrophoresis and EGFR, EGFRvIII and RCN1 expression was examined by western blot. (**B**) Whole cell lysates were also separated by gel electrophoresis and EGFRvIII and RCN1 expression was examined by western blot.

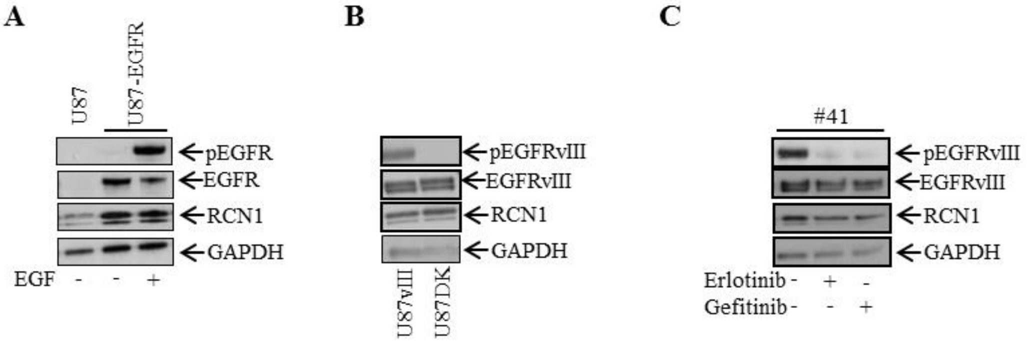

Figure S3. (**A**) U87 and U87-EGFR cells (stimulated with ± EGF; 100 ng/mL; 4 h) were lysed and assessed for pEGFR, EGFR, RCN1 and GAPDH expression by western blot. (**B**) U87vIII and U87DK cells were lysed and assessed for pEGFRvIII, EGFRvIII, RCN1 and GAPDH expression by western blot. (**C**) #41 cells were treated with ± Erlotinib or ± Gefitinib for 24 h then lysed and assessed for pEGFRvIII, EGFRvIII, RCN1 and GAPDH expression by western blot.

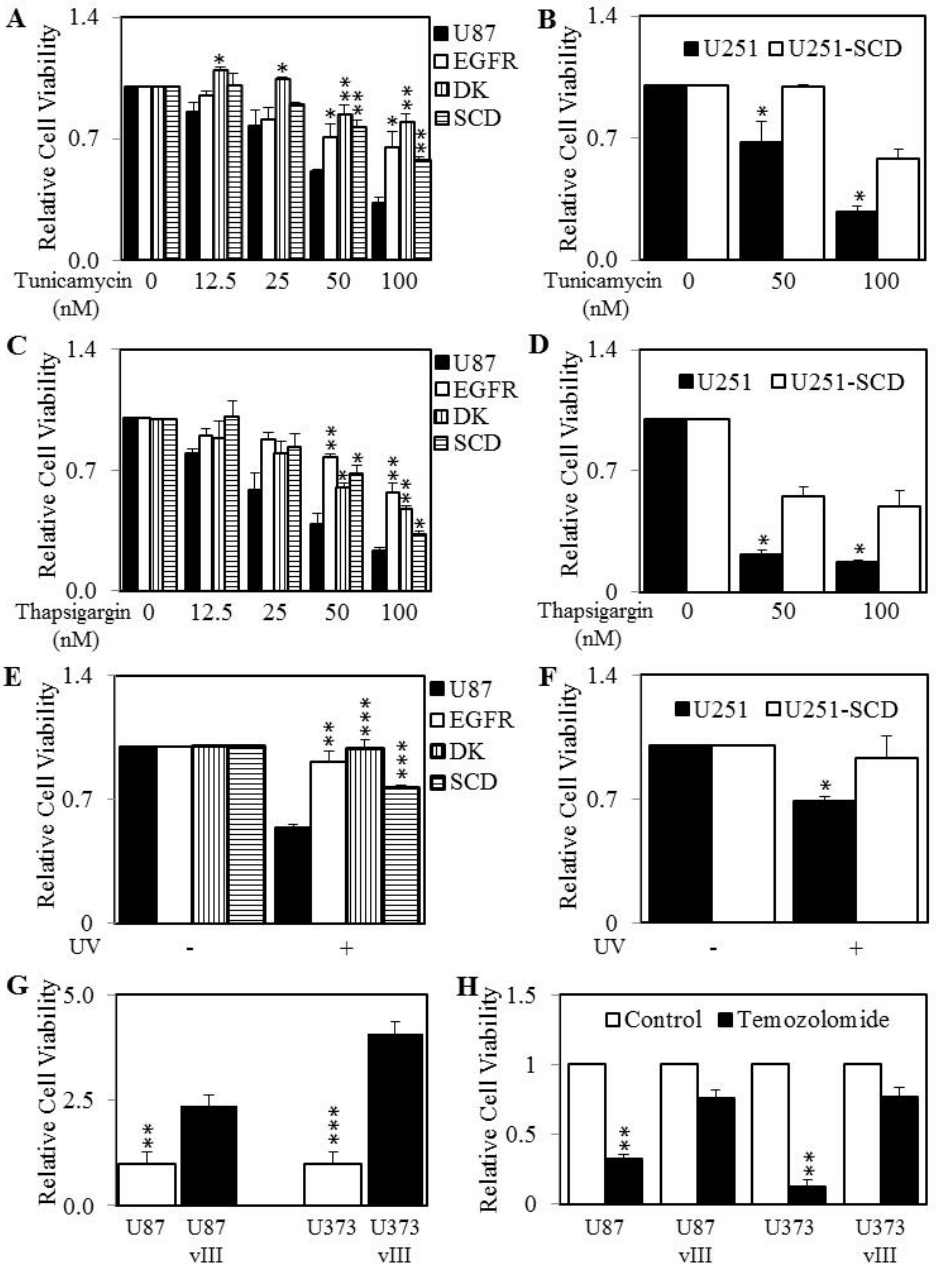

Figure S4. U87, U87-EGFR, U87DK, U87-SCD, U251 and U251-SCD cells were treated with increasing doses of tunicamycin (**A,B**) or thapsigargin (**C,D**) for 72 h. Cell viability was then determined using a commercially available Cell Titer-Glo kit and samples read on a bioluminometer. Data is expressed as % viability compared to untreated cells ± S.D of at least 3 independent experiments, each with 3 experimental replicates. U87, U87-EGFR, U87DK, U87-SCD, U251 and U251-SCD cells (**E,F**) were exposed to UV light for 10 mins and cell viability was examined following another 72 h as determined above. U87, U87vIII, U373 and U373vIII cells were seeded and allowed to adhere overnight. Media was then removed and replaced with (**G**) DME media free of glucose or H. media ± Temozolomide (2 mM) and cell viability was examined following 72 h as determined above. *$p < 0.05$; **$p < 0.01$; ***$p < 0.001$.

Fig 2A as appears in manuscript

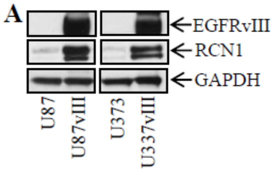

Original scans for Fig 2A

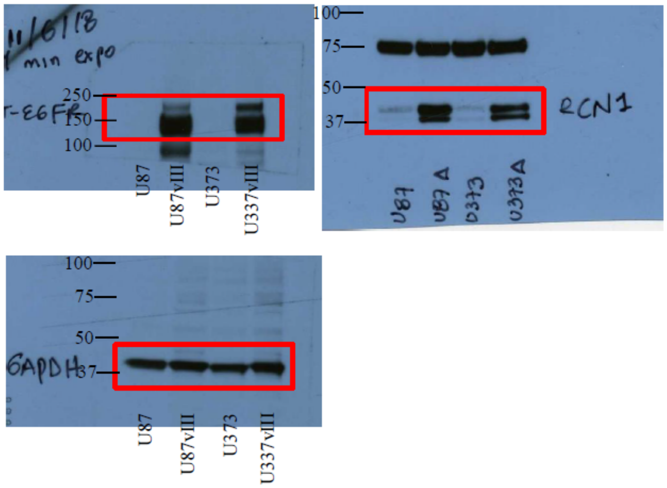

Fig 2B as appears in manuscript

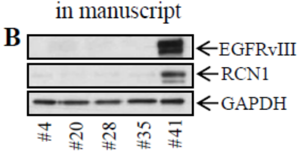

Original scans for Fig 2B

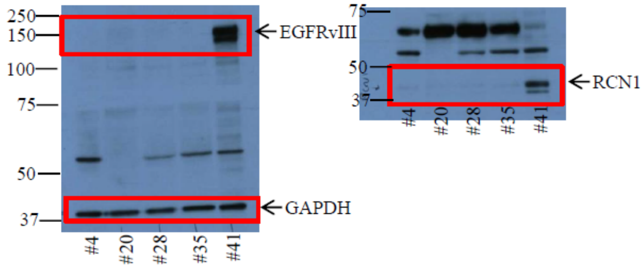

Fig 2E as appears in manuscript

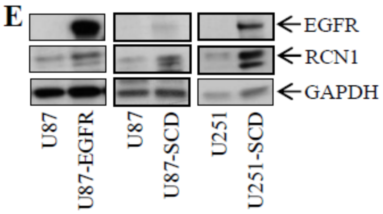

Original scans for Fig 2E

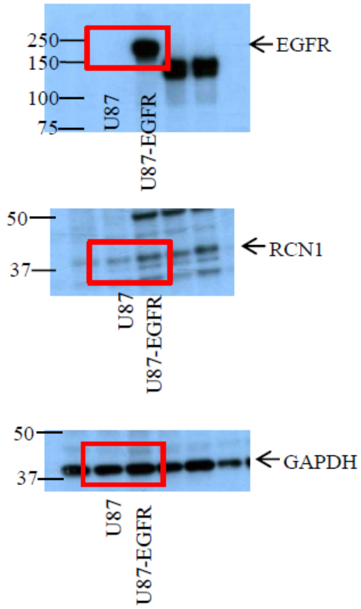

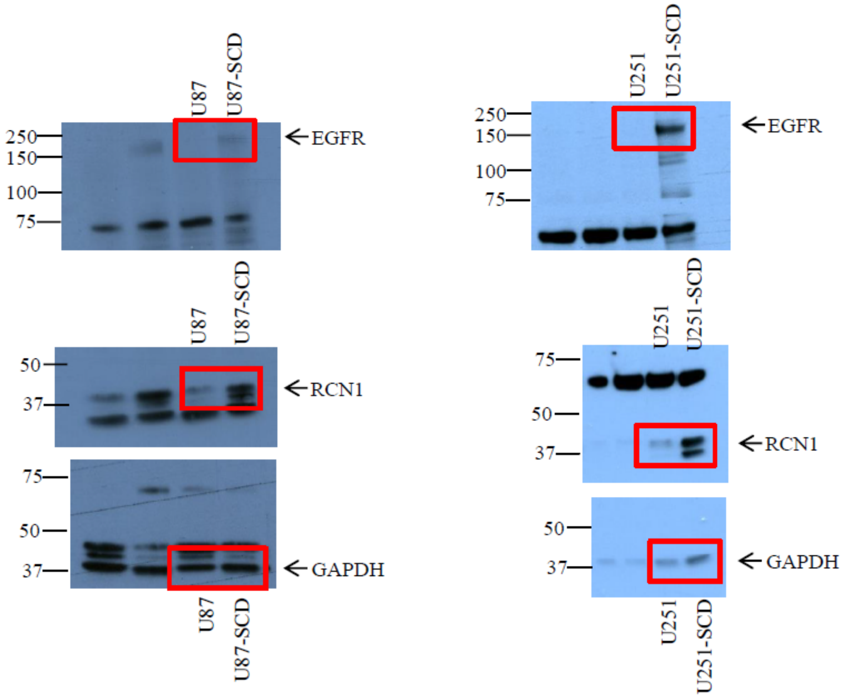

Detail information about Figure 2.

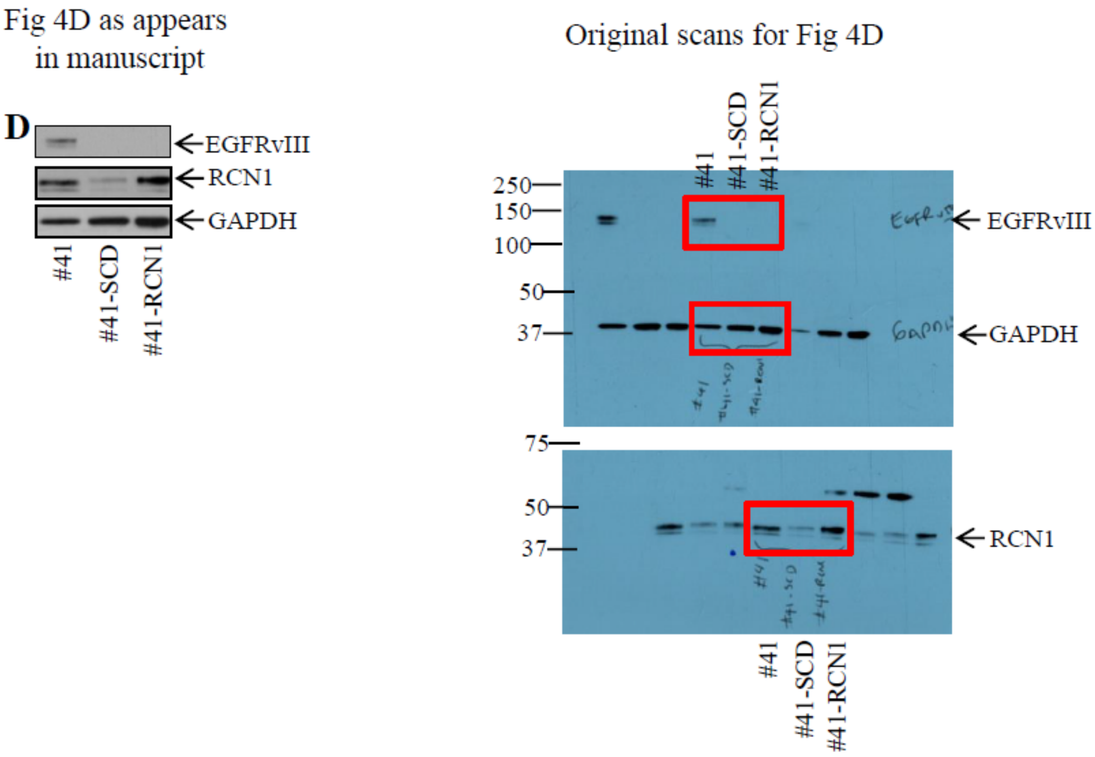

Detail information about Figure 4.

Fig 5G as appears in manuscript

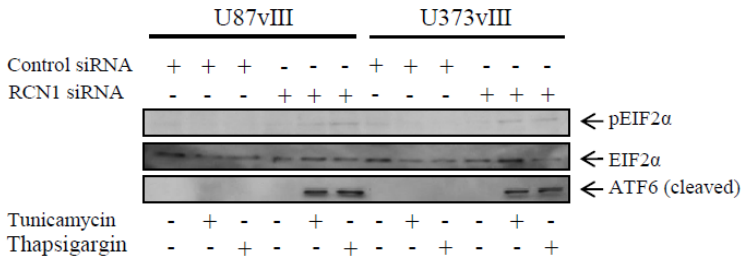

Original scans for Fig 5G

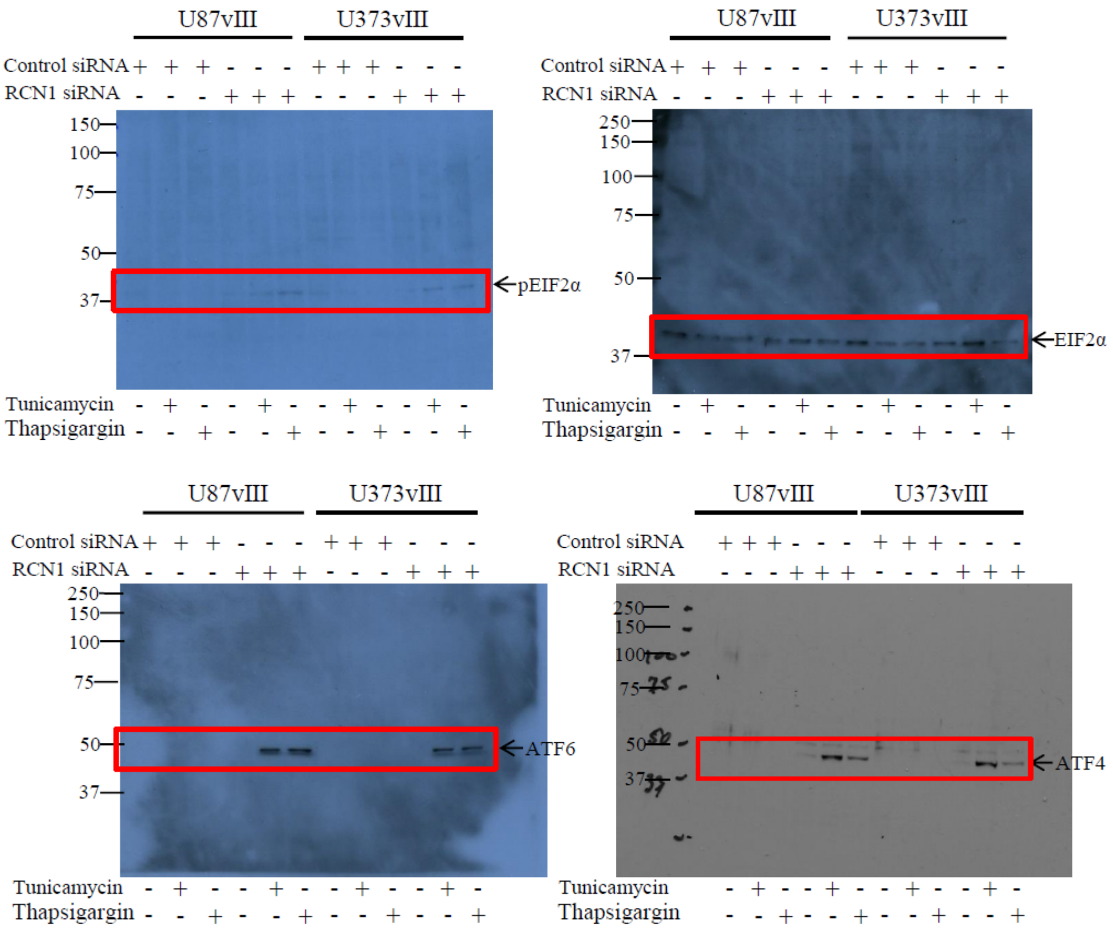

Detail information about Figure 5.

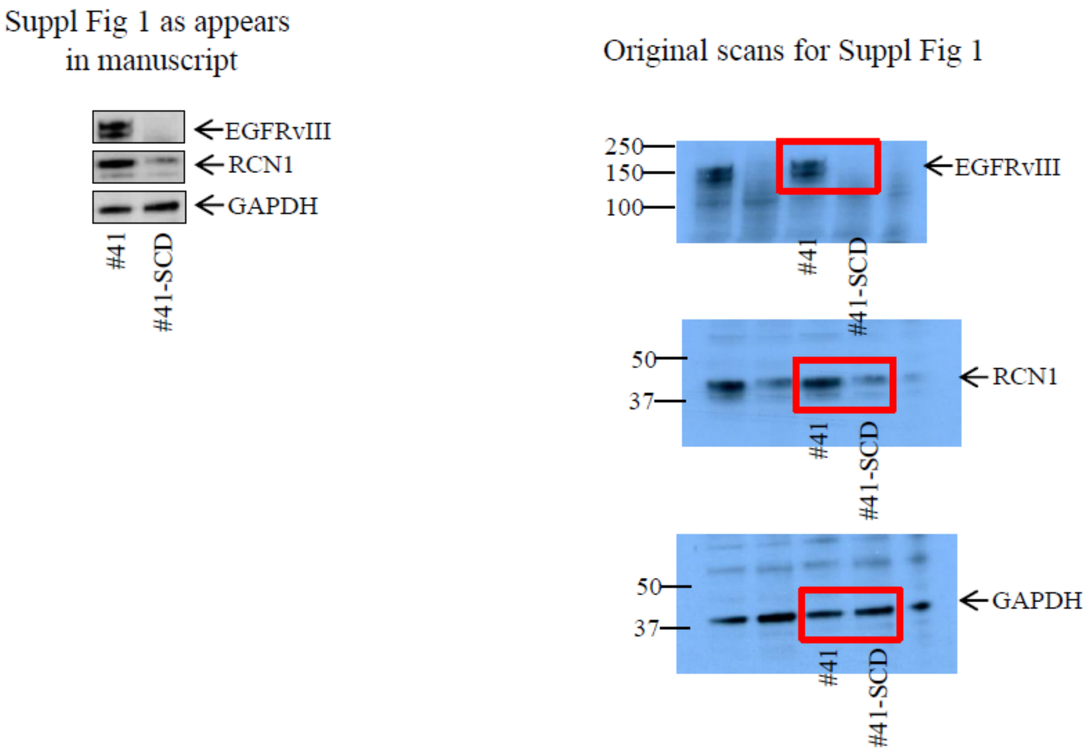

Detail information about Figure S1.

Suppl Fig 2A as appears
in manuscript

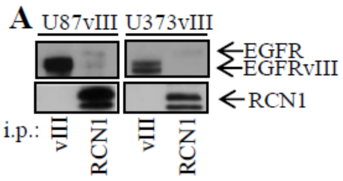

Original scans for Suppl Fig 2A

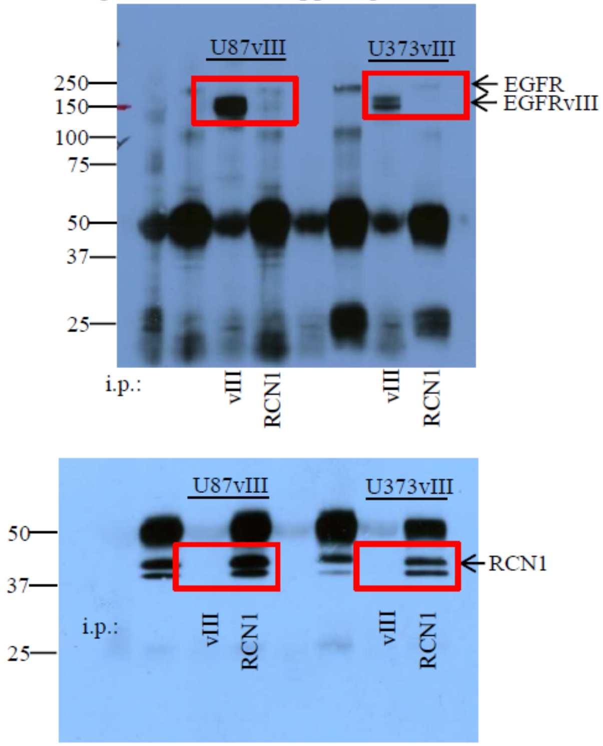

Suppl Fig 2B as appears
in manuscript

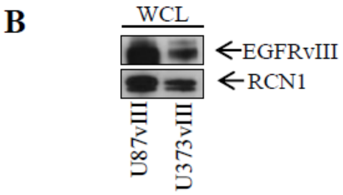

Original scans for Suppl Fig 2B

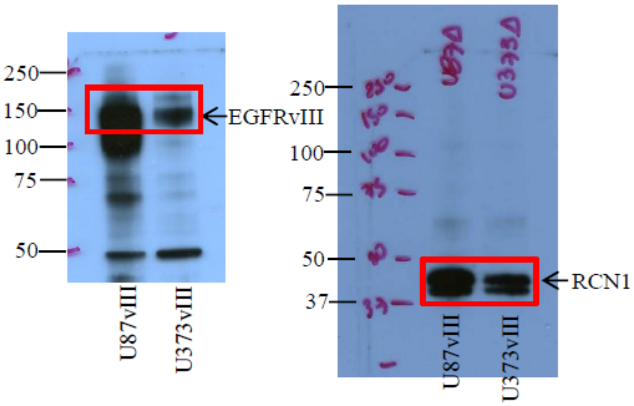

Detail information about Figure S2.

Suppl Fig 3A as appears in manuscript

A

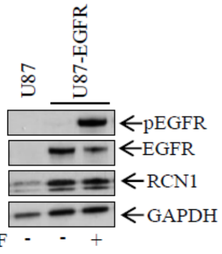

Original scans for Suppl Fig 3A

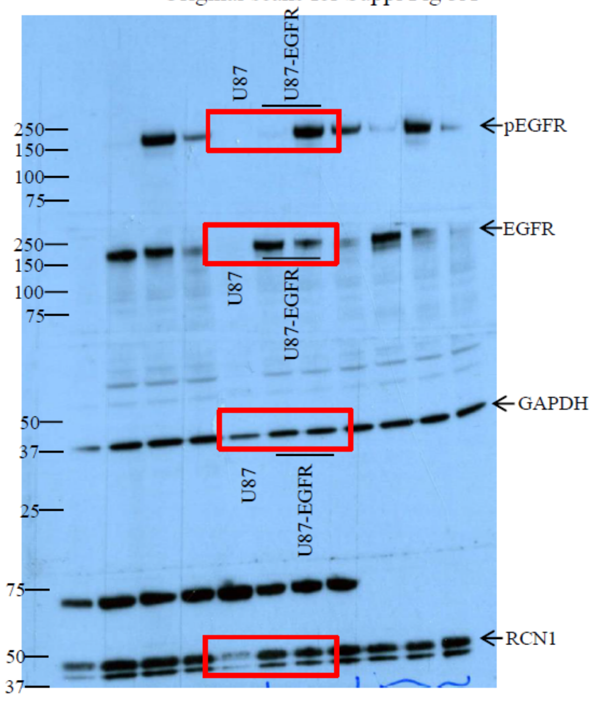

Suppl Fig 3B as appears in manuscript

B

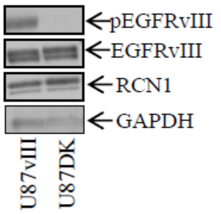

Original scans for Suppl Fig 3B

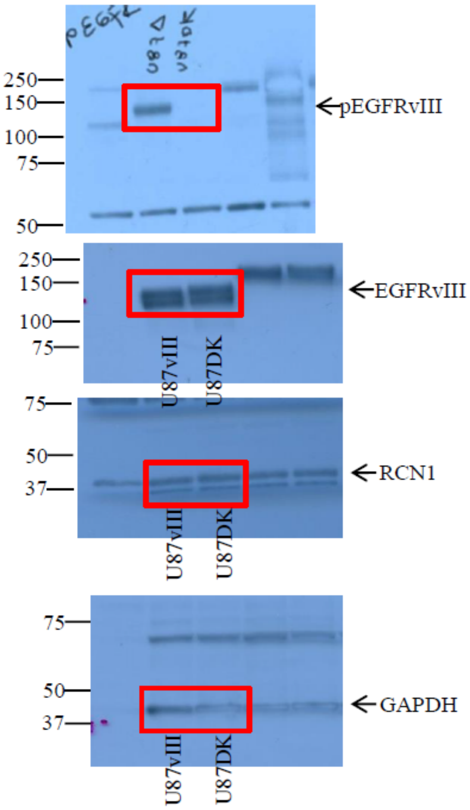

Suppl Fig 3C as appears in manuscript

Original scans for Fig Suppl 3C

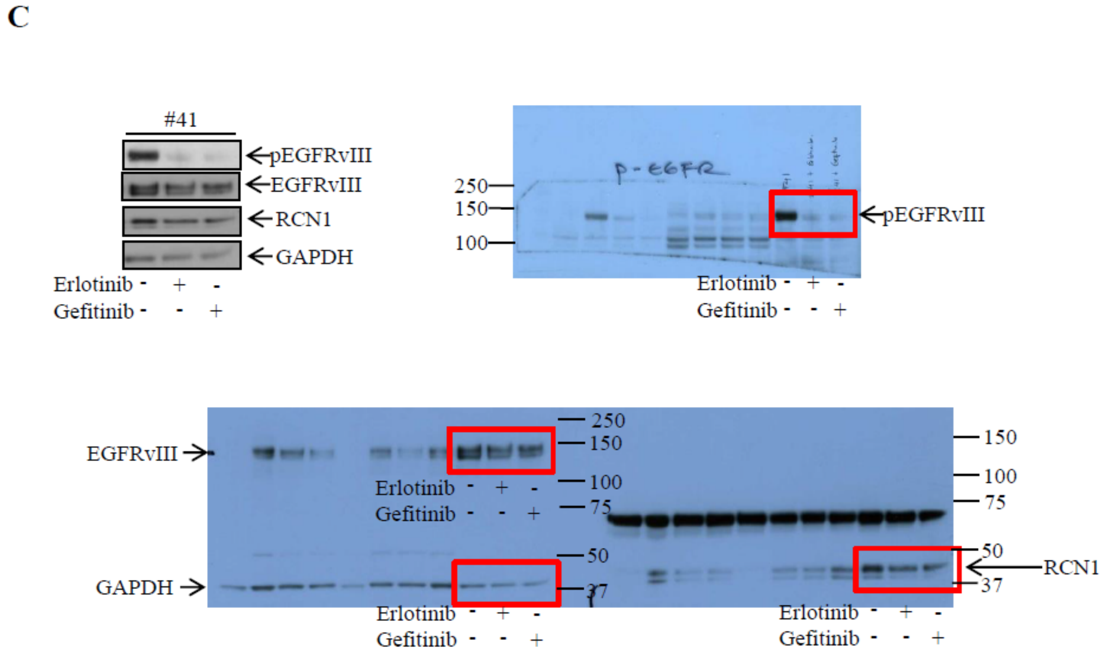

Detail information about Figure S3.

© 2021 by the authors. Licensee MDPI, Basel, Switzerland. This article is an open access article distributed under the terms and conditions of the Creative Commons Attribution (CC BY) license (http://creativecommons.org/licenses/by/4.0/).
